# Deep Assessment: A Novel Framework for Improving the Care of People with Very Advanced Alzheimer's Disease

**DOI:** 10.1155/2015/749451

**Published:** 2015-11-24

**Authors:** Gordon Lyons, Michael Arthur-Kelly, Ami Eidels, Aimee Mavratzakis

**Affiliations:** ^1^Centre for Special Education and Disability Studies, University of Newcastle, Callaghan, NSW 2308, Australia; ^2^School of Psychology, University of Newcastle, Callaghan, NSW 2308, Australia

## Abstract

Best practice in understanding and caring for people with advanced Alzheimer's disease presents extraordinary challenges. Their severe and deteriorating cognitive impairments are such that carers find progressive difficulty in authentically ascertaining and responding to interests, preferences, and needs. Deep assessment, a novel multifaceted framework drawn from research into the experiences of others with severe cognitive impairments, has potential to empower carers and other support professionals to develop an enhanced understanding of people with advanced Alzheimer's disease and so deliver better calibrated care in attempts to maximize quality of life. Deep assessment uses a combination of techniques, namely, Behaviour State Observation, Triangulated Proxy Reporting, and Startle Reflex Modulation Measurement, to deliver a comprehensive and deep assessment of the inner states (awareness, preferences, likes, and dislikes) of people who cannot reliably self-report. This paper explains deep assessment and its current applications. It then suggests how it can be applied to people with advanced Alzheimer's disease to develop others' understanding of their inner states and to help improve their quality of life. An illustrative hypothetical vignette is used to amplify this framework. We discuss the potential utility and efficacy of this technique for this population and we also propose other human conditions that may benefit from research using a deep assessment approach.

## 1. Introduction

According to the World Health Organization the number of people with dementia worldwide was estimated in 2010 to be 35.6 million and is projected to double every 20 years [1]. Global costs for treating and caring for people with dementia already exceed 600 billion US$ per year. Alzheimer's disease is the most common form of dementia and has no available treatment. It is therefore highly important to focus on advancing effective methods of caring.

Caring for and supporting people with advanced Alzheimer's disease (AAd) present extraordinary challenges to their family, carers, and other support professionals (hereafter referred to as “carers”) [2]. This is because, in addition to physical health decline, as their severe cognitive impairments impact functioning, patients often become increasingly difficult to communicate with and understand [3]. Therefore it becomes progressively more difficult for others to confidently ascertain and respond to changing interests, preferences, and needs [4].

Similar challenges face carers for people with comparable severe cognitive impairments like acquired brain injury and individuals with profound and multiple disabilities. For people with severe acquired brain injury there is generally an expectation that intervention will result in improved communication and understanding over time [5]. For people with profound multiple disabilities the challenges are life-long, but these people too can generally be expected to learn and develop improved communication skills over time [6]. Individuals with AAd, however, face a prognosis that includes an inevitable decline in communication skills and quality of life and eventually death. Carers struggle daily with this reality, as do people with AAd themselves [7].

The development of evidence-based practices in the care of people with Alzheimer's disease is the subject of considerable research activity [8]. Two of the most prominent and established approaches, Dementia Care Mapping [9] and Validation Theory [10], are good examples of evidence-based practices that have contributed to improved care and quality of life for those with Alzheimer's disease. These tools have informed personalized planning and the refinement of tailored supports in care programs. The goal of both techniques is to refine an understanding about the individual experience of Alzheimer's disease informed by the principles of ecological complexity in the provision of appropriate supports. Nevertheless, people with AAd present even more complex challenges to carers and continue to experience, by any measure, a comparatively poor quality of life [8].

By comparison, the lives of a significant proportion of people with severe acquired brain injury have improved due to technological improvements in medical treatment and therapy [5]. Furthermore, the lives of many people with profound multiple disabilities have improved in direct relationship to policy, practice, and attitudinal shifts amongst carers specifically [11] and the wider community in general [12]. The techniques of Behaviour State Observation [13], Triangulated Proxy Reporting [14], and Startle Reflex Modulation Measurement [15] are amongst the evidence-based techniques that have contributed to improved practices in these fields. Used in a harmonious way, we contend that these three techniques, collectively referred to as “deep assessment,” hold much promise for enhanced care and support for individuals with AAd.

Deep assessment is a novel multifaceted framework for delivering a more comprehensive and authentic assessment of the internal states of people with severe cognitive impairments who are unable to self-report. The term “severe cognitive impairments” arises from diverse sources including profound multiple disability, severe acquired brain injury, and advanced dementia. There is a history of using proxy reporters for people who are unable to self-report [21] but a continuing research agenda generally supports the view that simple proxy reporting lacks validity and accuracy [22]. This view is though contested [17] and research and policy around supported decision-making and substitute judgement are relevant here [23].

The next section describes the three components of deep assessment. The exposition is contextualized around current applications to people with profound multiple disabilities who, like their AAd counterparts, have severe cognitive impairments. They face significant challenges in communication and understanding of their inner states. This section is followed by a discussion about how deep assessment might be applied to persons with AAd, a group of persons who experience pervasive limitations in functioning due to severe cognitive impairments and communicative challenges. To illustrate this novel application, a hypothetical vignette is used to demonstrate in practical terms how deep assessment has, in the authors' opinions, the potential to inform carers about how to improve levels of understanding into various aspects of the internal states of people with AAd and consequently to improve both their quality of care and quality of life.

## 2. Methods

### 2.1. Deep Assessment

Generally, when we desire to know what other people think, the most reliable information comes from what they report or do. This includes overt behaviours such as facial expressions, body language, and verbal cues. However, this kind of information is limited if people cannot effectively or reliably provide this feedback, as is often the case with AAd patients [24]. This is also the case for infants and young children and people with severe communicative impairments allied with physical, cognitive, and/or emotional impairments. Sometimes it is necessary to ask others who know them well to provide their interpretation of this limited information.

People's inner states and how they are expressed are essential indicators of personal well-being, satisfaction, and subjective judgements on quality of life [25]. Understanding of these inner states informs daily and critical decision-making for carers [26]. Without this understanding, decisions about the care and well-being of others can only be based on secondary (proxy) reports informed mostly by notions of best interest [27] or by philosophical and moral judgement about what is judged to be right in any given situation [28].

At the heart of these debates is the question of veracity: how can we confidently know what a person wants if their wishes must be guessed by others? The deep assessment framework involves the strategic use of three complementary and synergistic assessment techniques that collectively deliver more robust data that is data less informed by “guessing.” Table 1 provides a summary of the techniques and their unique advantages.

These techniques are combined in an individualized manner to suit the needs of persons with specific severe cognitive impairments. The approach typically comprises a sequence of observations, reporting, and Startle Reflex Modulation Measurement, repeated frequently enough to maintain currency and authenticity in the light of any presumed or evidenced changes in internal states demonstrated by that individual. The techniques have significant application histories in the fields of special education for students with profound multiple disabilities [13], speech and communication therapy for people with severe communicative and cognitive impairments [29], and neuroscience/psychiatry [30].

By strategically combining these three techniques into the more comprehensive deep assessment framework, we hope to strengthen the veracity (rigor, validity, and reliability) of AAd caring protocols. Although these are not new techniques, their collective application to the populations represented in aged care, diversional therapy, and geriatrics is completely novel. A brief overview of each technique follows.

### 2.2. Behaviour State Observation

Carers need to communicate with those they support in order to best respond to their needs [31]. In most cases direct verbal dyadic interactions will suffice. In other situations where there are barriers to verbal communication (i.e., when a person cannot or will not interact directly with another in a verbal manner) direct observation is the next best source of information, followed by, rather than relying only on, proxy reports. Behaviour State Observation sits at the base of observational protocols and seeks to ascertain individual “readiness” or capacity to engage with environmental stimuli. Using finely grained categorical codes, judgements are made regarding both contextual variables such as sociocommunicative opportunities for interaction and the judged level of alertness in the target individual.

Behaviour State Observation originated in the pioneering work of Brazelton and Nugent [32] on infant behaviours and refers to various procedures for systematically coding levels of alertness and engagement in individuals, including those with profound and multiple disability [33]. The validity and reliability of these procedures, when used with people with severe cognitive impairments, continue to improve as ongoing research informs practice. Data is collected using paper and pencil techniques as well as video recording, supported by systematic interobserver checks. Variables observed in real time in addition to behaviour states include communication indicators and physical positioning.

Behaviour State Observation techniques span more than twenty years of research history, moving from descriptive data to sequential analyses and transitional probabilities, involving individuals with profound and multiple disability in a range of educational settings [33]. Critically, if carers can ascertain the “right moment” to engage with people with severe cognitive impairments, they are most likely to experience meaningful communications and consequently make informed care or educational decisions [34]. Generally, behaviour states are coded on a continuum from nonalert to most alert. Partial interval recording techniques, sometimes retrospectively analysed using video recordings, deliver a measure of the changes in levels of alertness and contextual variables. As communication partners become more skilled at reading changes in levels of alertness, they can strategically focus on those moments when the individual is in a most facilitative behaviour state to communicate with them and thence engage them in experiences which might bring them the most benefit or satisfaction. These judgements are informed by the social, communicative, and other contextual data collected simultaneously.

### 2.3. Triangulated Proxy Reporting

Triangulated Proxy Reporting involves the triangulation of data, investigators, and methodological approaches to analysis (see Denzin and Lincoln [35] for an extended explanation). The use of triangulation to strengthen data collection, analysis, and interpretation has a long history in both qualitative and quantitative research [35, 36]. There is less research on its formal application in authenticating and validating interpretations of attempts at communication made by people with severe communicative impairments in dyadic interactions (see Money [18] for an early application). The work of Lyons [17] in generating the Life Satisfaction Matrix, a procedure for ascertaining and improving the life satisfaction of people with profound multiple disabilities, is principally informed by Triangulated Proxy Reporting and serves as an example here.

In Lyons' approach two colleagues (who are familiar communication partners) observe the behaviours displayed (albeit usually idiosyncratically but generally consistently) by a focus person with severe cognitive impairments to express various inner states. They are then independently interviewed by a third (unfamiliar) colleague who records their observations.

A preliminary Behavioral Communication Profile is drafted, informed solely by observable discernable behaviours. Although the two colleagues may deliver a similar profile, consistency at this point is encouraging but is not a necessity. The behavioral descriptions offered by the familiar colleagues can often be clarified by analyzing naturalistic video recordings of those behaviours. Put simply, the Behavioral Communication Profile shows what the person does to show his/her range of feelings. Importantly, at this stage, the third colleague has not been told which daily experiences elicit these expressions of internal state.

The third colleague then observes the focus person during their routine day, building a familiarity with the Behavioral Communication Profile and looking opportunistically to record matches between these behaviours and daily experiences. This process should occur during regular daily activities, rather than during exceptional or irregular activities. Interviews are then repeated, this time reporting which daily experiences elicited which behaviours. If the third colleague is able to reasonably match the colleagues' reports, then, via the cross-referencing of the triangulation, the Behavioral Communication Profile is authenticated. Various daily experiences can then be confidently ranked in terms of their preferential appeal to the focus person. Different carers often engage with focus individuals in different daily experiences so ranking is unlikely to be highly similar across carers. However, observed dissimilarity in rankings does not detract from the evidence for the authenticity of the Behavioral Communication Profile itself. Furthermore, differences in views about the preferential appeal of different daily experiences can often be explained by conducting an activity or preference assessment similar to a task analysis (see Alberto and Troutman [37] for a detailed explanation) wherein differential preferences may be assigned to different components of a longer experience.

Triangulated Proxy Reporting functions to cross-check the knowledge and understandings of carers. It aims to produce a stronger collective confidence about the nature and purpose of observable behaviours displayed by individuals with severe cognitive impairments in the context of specific regular, naturalistic, and frequent daily experiences. Triangulated Proxy Reporting techniques have an extensive research history (see Lyons [17]). If carers can authenticate their knowledge about how people with severe cognitive impairments express their inner states and rank the relative preferential appeal of daily activities, then the carers can make informed care decisions. Like Behaviour State Observations, these techniques function as a secondary source of data that relies on intensive collaborative consultations and observations. Therefore, deep assessment proposes the inclusion of a third technique to enhance its rigor: Startle Reflex Modulation Measurements.

### 2.4. Startle Reflex Modulation Measurement

Thus far, the paper has described two techniques that have a substantial subjective element. In contrast, Startle Reflex Modulation Measurement (SRM) provides an objective measurement of individuals' inner states. In neuroscientific research, psychophysiological measures such as SRM, heart beat rate, breathing rate, and skin conductance have long been considered objective measures of basic emotional states because they provide direct and fast indications of emotional reactions that are typically obscured by higher cognitive processes [38]. In particular, SRM is considered the only objective physiological measure of emotion that is sensitive to emotional valence; that is, it can tell apart negative and positive internal emotional states [19]. When used within the deep assessment framework, SRM can complement and enhance assessments made using Behaviour State Observations and Triangulated Proxy Reporting. More importantly, as a measure of internal emotional states, SRM can provide authentic personalized information to empower carers of people with severe cognitive impairments, allowing them to make more informed decisions.

An advantage of SRM is its strong statistical properties. Psychophysiological measures face fewer criticisms around validity and reliability than measures based on behavioral observations and self-reporting and proxy reporting. The early work of Freeman et al. [39] demonstrated that physiological measures enhance understanding of the life experiences of people with severe cognitive impairments. More recent work by Vos et al. [40] showed that changes in some physiological measures correlated with changes in the perceived emotional states of people with profound multiple disabilities.

Most psychophysiological measures can indicate changes in the levels of arousal and can augment Behaviour State Observation. However, such measures are by and large not indicative of changes in emotional valence [19] because preferred (pleasant) and nonpreferred (unpleasant) experiences can deliver very similar measures of arousal. The SRM measurement technique, on the other hand, is unique due to its sensitivity to emotional valence (or affect) and has been argued to be even more sensitive to emotional states relative to self-reporting or proxy reporting [20]. SRM therefore has the potential to deliver a reliable measure of emotional valence (degree of experienced pleasantness) in terms of simple motivational preferences without requiring explicit responses. For people unable to communicate due to declining cognitive functioning, such as people who suffer AAd, SRM may be a window (perhaps the only known reliable window) into inner emotional states and preferences.

SRM is based on electromyographic data derived from differential eye blink amplitudes to distinguish between positive (pleasant) and negative (unpleasant) emotional internal states precipitated out of a diversity of sensory experiences [30]. SRM is a product of the startle reflex, an evolved motivational-based adaptation common to all complex living organisms that involves a spontaneous and involuntary full-body muscle contraction in response to a sudden and unexpected stimulus, allegedly intended to quickly and reflexively withdraw the organism from potential harm. The startle reflex may be elicited by tactile or visual sensory stimulations, but most commonly by a sudden, fast-rising auditory stimulus. The strength of the startle response and more specifically the strength of the associated eye blink provide an indication of the organism's current inner emotional state.

**Figure 1:**
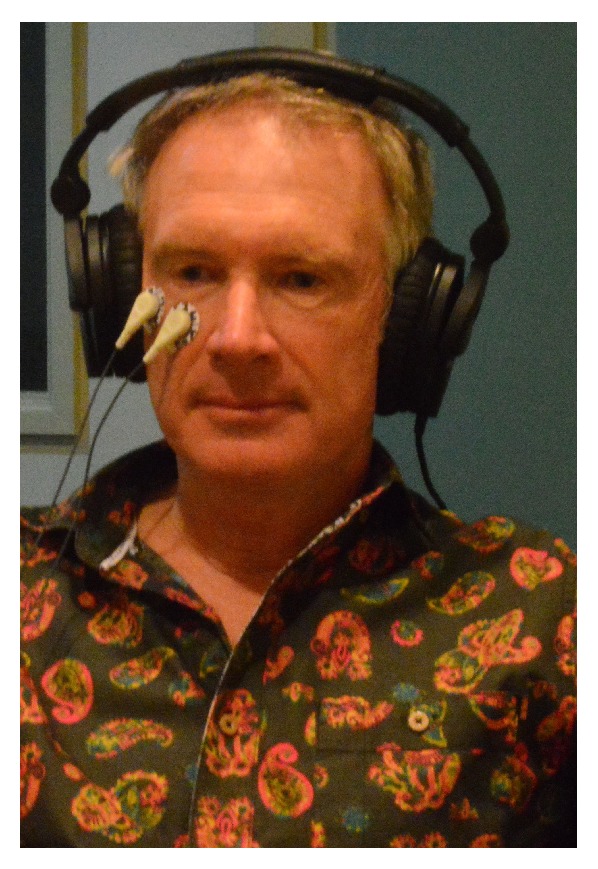
The SRM apparatus attachments.

The robustness of the startle-associated eye blink provides a reliable index of relative emotional valence such that a stronger eye blink indicates a more negative or unpleasant current inner state, whereas a weaker eye blink indicates a more positive or pleasant inner state [41].  Figure 1 illustrates the simple apparatus used to collect the SRM data streams. The testing apparatus and procedure are only minimally intrusive. In addition to the headphones, two wired adhesive pads attached to the individual's face and a discreet grounding pad are the only attachments. Once the calibration of the individual's readings is completed, attachment time is minimal for each stimulus test.

SRM is an objective measure that requires no conscious appraisal or language-related responses and is independent of cognitive processing and arousal. This is because the primary neural circuits involved in the eye blink component of the reflex occur in primitive subcortical regions of the brain that process emotional information at a basic motivational level, where simple approach or avoidance behaviours are generated, largely independent of higher cognitive function [38].

In terms of feasibility of the approach, measuring the startle eye blink reflex has been demonstrated in people with Alzheimer's disease [42] primarily for the purpose of understanding changes in brain structure and function related to the disease. SRM protocols have also been used across diverse clinical populations in neuroscientific and psychological research to cross-check the validity and reliability of self-reported emotional responses and behaviours (for reviews see [43, 44]). Current recommendations are that caregivers should develop nonverbal approaches to communicating with individuals with pervasive cognitive impairments in anticipation of losing the ability to communicate using language [45]. SRM measurement is ideal for this purpose, particularly when conducted in the broader context of a multifaceted deep assessment framework that allows for the utilization and comparison of multiple patient and caregiver data inputs.

## 3. Discussion

The techniques of Behaviour State Observation, Triangulated Proxy Reporting, and Startle Reflex Modulation have substantial independent histories in other fields of research and practice, but only the latter has found some traction in the field of gerontological research [45]. Deep assessment combines the three techniques into a novel, comprehensive assessment framework that benefits from two major considerations.

First, researchers and practitioners in the fields of biomedical research, aged care nursing, gerontology, and diversional therapy have not, to the best of our knowledge, connected these techniques. The research and practice fields of special education, intellectual disability education, aged care, and neuropsychology rarely cross paths so it is not surprising that these techniques have lacked collaborative investigations. Multidisciplinary engagement amongst researchers and practitioners is lauded and encouraged at a systemic level [46] but does not occur so often in practice because pilot funding for encouraging basic and applied action research around untried and untested (novel) theory and practice is hard to attract, and, in the social sciences, ethics hurdles may be insurmountable.

Second, care and support planning for other populations with severe cognitive impairments is generally developmental and intervention focused. That is, for people with severe acquired brain injury, planning is usually focused on partial recovery, rehabilitation, and restoration of functionality. For people with profound multiple disabilities, education and therapy are usually focused on identifying and developing skill and ability potentials. In something of a contrast, for people with AAd, care and support planning is generally focused on minimizing the unavoidable deleterious effects of advancing cognitive and physical decline.

Furthermore, this decline in people with AAd is predictable only in that it is unavoidable. On any one day and at any one time, people with AAd can present with very inconsistent apparent interests and preferences, although there are some predictability and patterns to prevailing behavioral problems [47, 48]. Substitute decision-making is fraught with challenges. On the one hand many people with AAd have significant histories of interests and preferences. These can change gradually, as they do for most people, or very quickly and unpredictably, and these changes can be temporary and/or fluctuating or sustained. Any assessments of behaviour state and inner state need to be repeated as often as possible, to maximize the probability that substitute decision-making enhances quality of care and improves the quality of life.

The following vignette illustrates how deep assessment might be operationalized to benefit May (pseudonym), a person with AAd. The vignette illustrates how the approach might be applied in a typical care scenario to maximize May's quality of care and quality of life.

## 4. A Hypothetical Vignette

May is an 86-year-old widow. She has lived in an aged care facility for three years. She is visited usually twice a week by Mary, her daughter, monthly by Bill, her son, and very occasionally by various grandchildren. May has had Alzheimer's disease for at least six years. At the time of moving into the facility she was physically frail, unable to ambulate, and incapable of caring for herself safely. She was also showing clear signs of midstage dementia with near-daily episodes of disorientation, confusion, and agitation.

May was previously a happy, active, and socially engaging person. Yet her Alzheimer's disease had now progressed to the stage wherein she was most often nonalert, “dozing,” or asleep, incommunicative, and primarily unresponsive to verbal and even physical prompts by her family and care staff. Her quality of life, by any measure, was poor and was deteriorating, and both the familiar care staff and her children were now unable to consistently or confidently determine what May wanted or did not want, preferred or did not enjoy, or even needed (both socially and emotionally). With a continuing trajectory of physical, intellectual, and emotional decline the only certainty for those supporting May was to address her high physical care needs. There had been familial discussions around the apparent futility and “senselessness” of May's life but Mary and Bill and the care staff still tried to bring some enjoyment to Mary by attempting to engage her in reminiscent conversation and sensory experiences that she had historically enjoyed.

After some discussions between Mary, Bill, and key care staff, it was decided to conduct a deep assessment. Diminishing quality of life was clearly the primary concern and it was hoped that deep assessment might offer more informed and authentic insights into May's difficult-to-discern interests and preferences and into her levels of alertness so that needs and desires might be better responded to.

The facility's diversional therapist first conducted a set of Behaviour State Observations on May. These suggested a “topography” or profile of alertness indicators and changes that were not evident through informal observations or indicated through historical proxy knowledge. At the same time codes were recorded about communication partners, interactions, and social grouping observed at any one time. This information helped key staff and family to identify and target key periods and moments in May's day when she was more likely to be receptive to communicative and potentially enjoyable engagements orchestrated by others. For example, though May was consistently unresponsive to background noise, when someone played a piano nearby her entire observed state altered and she was generally more responsive to the communication cues of those around her.

Around the same time key care staff and family “catalogued” and ranked those activities and experiences that had historically and most recently brought a positive emotional response from May. Using the Triangulated Proxy Reporting technique a reasonably confident consensus was reached concerning May's preferred and less preferred experiences. This analysis was complemented by a list of “new” experiences that care staff and family brainstormed to explore using Startle Reflex Modulation Measurement. The diversional therapist conducted two sessions of SRM measurements: first to initially calibrate the affect measurements and second to determine the affect potential of some of the “new” experiences put forward by care staff and family.

A “quality of life enhancement plan” was then drafted from a collaborative analysis of the data provided by the deep assessment. The gist of this plan was shared amongst the care staff and family members. May's overall care taking and daily schedule were modified to reflect and respond to the plan. Although the measures taken are not likely to extend May's life expectancy, they will improve the quality of her life, as well as that of a wider circle of people who surround her, such as family members and the facility's caregivers.

How might this approach differ from more traditional approaches to assessing May's support priorities? First, it involves potential input from a larger number of key participants in May's social ecology as well as May herself, though not via explicit verbal or motor directions. Second, data collected across time and modes enhances the internal validity of any conclusions drawn for planning purposes. Lastly, such a comprehensive approach avoids the dyadic “clinician and May” approach that still dominates traditional models of support.

## 5. Conclusions

Deep assessment is a new and innovative framework for empowering families, carers, and other support professionals to better understand and make improved care decisions for people with advanced Alzheimer's disease. Its component parts (Behaviour State Observation, Triangulated Proxy Reporting, and Startle Reflex Modulation) are also novel to this specific field of Alzheimer's disease but have substantial research histories in other areas of research.

The techniques within deep assessment have already demonstrated potential to inform and empower family and carers to enhance the quality of care and ultimately the quality of life of persons with profound multiple disabilities, who historically experienced a relatively low quality of life. It has been argued that it also has potential to inform and empower the family and carers of other persons with severe cognitive impairments, particularly those with advanced Alzheimer's disease. Persons with Alzheimer's disease constitute an expanding population in the world's aging society, and as advances in biomedical research and care practices continue to extend the average life expectancy, governments and society need to respond in a range of ways that will include moral, philosophical, and fiscal dimensions. We argue that there is a clear social justice imperative to find better ways to improve the diminishing quality of life of an increasing number of people who live with advanced Alzheimer's disease. In this context the authors have a commitment to drive a research agenda that aims to explore the potential of deep assessment for people with advanced Alzheimer's disease. Deep assessment represents an exciting opportunity in multidisciplinary collaborative research, generally for people with severe cognitive impairments and specifically for people with advanced Alzheimer's disease.

Notwithstanding challenges with respect to funding and ethical aspects in such a complicated but important area, the authors conclude by calling for interdisciplinary collaborations with colleagues in neuroscience, biomedicine, aged (nursing) care, geriatrics, and/or diversional therapeutics to launch this neophyte research agenda. As a starting point, pilot trials utilizing deep assessment with a small and relatively homogeneous sample may serve to inform practical and methodological considerations. These can then serve as a basis for larger scale investigations utilizing control groups and stratified participant groups as a means of testing its efficacy in a robust manner. In effect, this comprehensive approach also holds promise for other subgroups in the aging population, including individuals with generalized dementia and stroke-induced communication challenges.

## Figures and Tables

**Table 1:** Summary of the three techniques comprising deep assessment.

Technique	Description	Advantages	Key refs.
Behaviour State Observation	An observational protocol that uses fine-grained category codes to allow judgements about a person and their social and communicative contexts.	A detailed and systematic means of connecting individual levels of alertness and engagement with a range of relevant contextual variables, including sociocommunicative elements.	[15, 16]

Triangulated Proxy Reporting	A systematic consultative process drawing on the close personal knowledge of individual needs as displayed in a range of natural settings.	It allows for the authentic and powerful input of those who know the person best. It allows the direct translation of carer knowledge into planning and support for the person with participation challenges.	[17, 18]

Startle Reflex Modulation	An electrophysiological measurement of emotion. The amplitude of a person's reflexive eye blink in response to a startling stimulus indicates whether the individual is experiencing a more unpleasant or a more pleasant inner state.	It allows unbiased and implicit measurement, not requiring verbal responses. A reliable indicator of basic emotional preference even for a person in cognitive decline.	[19, 20]
